# Could Dupilumab Improve Sleep Quality in CRSwNP Patients: Myth or Reality? A Systematic Review

**DOI:** 10.3390/jcm15156010

**Published:** 2026-08-02

**Authors:** Antonio Moffa, Eugenio De Corso, Domiziana Nardelli, Ahmed Yassin Bahgat, Antonella Loperfido, Iman Al Afifi, Ewa Olszewska, Peter M. Baptista, Jacopo Galli, Manuele Casale

**Affiliations:** 1 Department of Otolaryngology, Fondazione Policlinico Universitario Campus Bio-Medico, 00128 Rome, Italy; a.moffa@policlinicocampus.it (A.M.); i.alafifi@gmail.com (I.A.A.); m.casale@policlinicocampus.it (M.C.); 2 School of Medicine, Università Campus Bio-Medico di Roma, 00128 Rome, Italy; 3 Section of Otorhinolaryngology, Department of Medicine and Surgery, Santa Maria della Misericordia Hospital, 06156 Perugia, Italy; eugenio.decorso@unipg.it; 4 Otorhinolaryngology-Head and Neck Surgery, University of Perugia, 06132 Perugia, Italy; 5 Saudi German Hospitals, Dubai 391093, United Arab Emirates; ahmedyassinbahgat@gmail.com; 6 Otolaryngology Unit, San Camillo Forlanini Hospital, Circonvallazione Gianicolense 87, 00152 Rome, Italy; aloperfido@scamilloforlanini.rm.it; 7 Department of Otolaryngology and Head and Neck Surgery, Al Nahda Hospital, Ministry of Health, Muscat 113, Oman; 8 Department of Otolaryngology, Sleep Apnea Surgery Center, Medical University of Bialystok, 15-276 Bialystok, Poland; ewa.olszewska@umb.edu.pl; 9 Otorhinolaryngology Department, Clinica Universitaria de Navarra, 31008 Pamplona, Spain; peterbaptista@gmail.com; 10 Department of Head-Neck and Sensory Organs, Università Cattolica del Sacro Cuore, 00168 Rome, Italy; jacopo.galli@policlinicogemelli.it; 11 Unit of Otorhinolaryngology-Head and Neck Surgery, A. Gemelli Hospital Foundation IRCCS, 00168 Rome, Italy

**Keywords:** dupilumab, chronic rhinosinusitis with nasal polyps (CRSwNP), sleep quality, type 2 inflammation, patient-reported outcome measures (PROMs), SNOT-22, pittsburgh sleep quality index (PSQI), obstructive sleep apnea (OSA)

## Abstract

**Background/Objectives**: Chronic rhinosinusitis with nasal polyps (CRSwNP) is a type 2 inflammatory condition that significantly impairs health-related quality of life (HRQoL), particularly sleep quality. Beyond mechanical nasal obstruction, type 2 cytokines (IL-4, IL-13) are thought to directly disrupt sleep architecture. Dupilumab, an IL-4Rα antagonist, is approved for severe CRSwNP, but its specific effect on sleep quality remains under investigation. This systematic review aims to evaluate the impact of dupilumab on sleep quality in patients with severe, uncontrolled CRSwNP. **Methods**: A comprehensive literature search was conducted in PubMed/MEDLINE, Google Scholar, and Web of Science up to June 2026. We included adult studies (≥1 month of dupilumab 300 mg every 15 days) reporting sleep outcomes using validated tools (Epworth Sleepiness Scale [ESS], Pittsburgh Sleep Quality Index [PSQI], Insomnia Severity Index [ISI], or the SNOT-22 sleep domain). Risk of bias was assessed using ROBINS-I and RoB 2 tools. **Results**: Seven studies (*n* = 2164 patients) met inclusion criteria. Across observational and post hoc RCT analyses, dupilumab consistently improved the SNOT-22 sleep domain (mean reduction up to −7.02 points at 24 weeks; *p* < 0.001), PSQI, ESS, and ISI scores. One study reported a decrease in poor global sleep quality from 88.9% at baseline to 5.7% at 12 months. However, most observational studies had a serious risk of bias due to unaddressed confounding and lack of blinding. No polysomnographic data were reported. **Conclusions**: Dupilumab was associated with significant improvements in patient-reported sleep quality in CRSwNP patients across seven included studies. However, the evidence is limited by the absence of objective sleep measures, the serious risk of bias in most observational studies, and the lack of comparative head-to-head data. High-quality randomized controlled trials with prespecified sleep endpoints and polysomnographic assessments are needed before definitive conclusions can be drawn.

## 1. Introduction

Chronic Rhinosinusitis is a multifactorial inflammatory condition with two clinical phenotypes: chronic rhinosinusitis without nasal polyps (CRSsNP) and with nasal polyps (CRSwNP). CRSwNP represents 20% of cases, characterized by the development of benign, oedematous nasal polyps originating from the ethmoid sinuses and extending into the nasal cavities [1]. This phenotype is mainly caused by type 2 inflammation, associated with a type 2 cellular response, type 2 cytokine production (IL-4, IL-13, and IL-5), and increased tissue and serum IgE levels [2]. Patients with CRSwNPs experienced a profound impact on their Health-Related Quality of Life (HRQoL). A recent patient survey reported that CRSwNP patients experience very bothersome symptoms, such as nasal congestion, headache, rhinorrhea, and frequent sleep disturbances, leading to an average of 24.7 missed workdays per year. Moreover, the patients and their caregivers showed significantly lower sleep quality, experiencing poor nights’ sleep on average 72.1 and 51.7 days per year, respectively [3].

In particular, it is known that CRSwNP affects sleep architecture and nocturnal QoL. It is very common for those patients to suffer from insomnia and an increased risk of Sleep-Disordered Breathing (SDB), including Obstructive Sleep Apnea (OSA) and poor sleep quality, which, in turn, is significantly correlated with decreased daily work productivity, daytime fatigue, and cognitive dysfunction. According to the European Position Paper on Rhinosinusitis and Nasal Polyps EPOS/EUFOREA 2023 update guidelines, CRSwNP and sleep disturbances represent a significant burden on QoL, underlining the need for optimized treatment strategies for these patients, as the global quality of sleep and life is compromised [4].

The mechanisms linking CRSwNP to sleep disorders are multifactorial. Nasal congestion and airflow obstruction are the main contributing factors, as increased upper airway resistance disrupts normal breathing during sleep, reduces blood oxygen saturation, and leads to poor sleep efficiency, fragmented sleep, and excessive daytime sleepiness [4,5]. However, new research suggests that type 2 inflammation, which characterizes CRSwNP, plays a crucial role in sleep physiology disruption, revealing that the cause is not only mechanical [5]. Addressing sleep impairment has become an important therapeutic goal, often passing through effective management of airway inflammation and nasal obstruction, contributing to better sleep quality and overall well-being. For instance, the impact of CRS treatments on sleep quality has been systematically evaluated beyond the specific context of biologic therapy. Fried et al. [6] conducted a systematic review and meta-analysis of 16 studies comprising 1770 patients, examining the effect of conventional medical and surgical CRS treatments on subjective and objective sleep metrics. They found clinically meaningful improvements in patient-reported sleep outcomes, including the Epworth Sleepiness Scale (ESS), Pittsburgh Sleep Quality Index (PSQI), and the SNOT-22 sleep domain. However, objective sleep parameters, specifically the AHI and oxygen nadir, did not demonstrate corresponding improvements. This dissociation between subjective and objective sleep measures raises important questions about the mechanisms linking upper airway inflammation to perceived sleep quality.

Recent evidence suggests that biologic therapies targeting type 2 inflammation, such as dupilumab, may improve sleep patterns in CRSwNP patients, likely by reducing inflammation-driven nasal congestion and enhancing nocturnal breathing comfort. Dupilumab is a fully humanized monoclonal antibody that targets the IL-4 receptor alpha (IL-4Rα), effectively inhibiting IL-4 and IL-13 signaling, thereby reducing type 2 inflammation, eosinophilic infiltration, and restoring epithelial barrier function. It is the first monoclonal antibody medication approved by the US Food and Drug Administration (FDA) for treating CRSwNP. Recent real-world evidence has further consolidated the efficacy of dupilumab in CRSwNP. A systematic review with meta-analysis by Rodriguez-Iglesias et al. [7] synthesized data from 26 real-world studies comprising 2183 patients, demonstrating a mean SNOT-22 reduction of 37.2 points and a mean Nasal Polyp Score (NPS) reduction of 3.6 points following dupilumab treatment: improvements that exceeded those reported in the pivotal SINUS-52 randomized controlled trial. These findings underscore the robust effectiveness of dupilumab in clinical practice; however, the specific impact on sleep quality, a domain consistently identified as one of the most severely affected by CRSwNP, was not the primary focus of that analysis and warrants dedicated investigation.

Given the strong link between sleep disturbance and overall disease burden in CRSwNP, this systematic review aims to evaluate the effects of dupilumab on sleep quality, investigating its potential to improve nocturnal respiratory function and overall sleep-related well-being in patients with severe and uncontrolled CRSwNP.

## 2. Materials and Methods

This review was conducted in accordance with the Centre for Reviews and Dissemination guidance for undertaking reviews in healthcare and is reported in line with the Preferred Reporting Items for Systematic Reviews and Meta-Analyses (PRISMA) 2020 statement [8].

### 2.1. Data Source and Study Searching

A comprehensive literature search was performed in the main biomedical databases, including PubMed/MEDLINE, Google Scholar, and Web of Science. The search strategy combined Medical Subject Headings (MeSH) and free-text terms related to chronic rhinosinusitis with nasal polyps, dupilumab, and sleep quality. An example of a search strategy is the one used for PubMed/MEDLINE: “Dupilumab” and “Chronic Rhinosinusitis”; “Dupilumab and “CRSwNPs and “sleep”; “Dupilumab” and “Sleep”; “Dupilumab” and “Sleep effects”; “Dupilumab” and “SNOT-22”; “Dupilumab” and “ESS”; “Dupilumab” and “PSQI”; “Dupilumab” and “Real life”. The search strategy was adapted to the specific requirements of each database. In addition, manual cross-referencing of the bibliographies of all included articles was performed independently by two reviewers to minimize the risk of missing relevant studies. Systematic reviews and guidelines were excluded to prevent duplication of data and potential bias from overlapping study populations. By focusing on primary studies, this review aimed to provide a more granular and critical interpretation of the available evidence, particularly in a rapidly evolving field such as biologic and surgical integration in CRSwNP. The final search was conducted in March 2026.

### 2.2. Inclusion/Exclusion Criteria

We included the studies with the following characteristics: patients (P), adult patients affected by severe CRSwNPs defined by a nasal polyp score (NPS) ≥ 5 and/or a Sinonasal Outcome Tests-22 (SNOT-22) ≥ 50, with inadequate symptom control despite intranasal corticosteroids (INCS) use, receiving at least two cycles of systemic corticosteroid in the last year and/or having undergone one or more endoscopic sinus surgery; intervention (I), at least 1 months of biological therapy administration (Dupilumab 300 mg, one subcutaneous injection every 15 days) indicated specifically for severe CRSwNP treatment; comparison (C), pretreatment and post-treatment; outcome (O), Epworth Sleepiness Scale (ESS), Pittsburgh Sleep Quality Index (PSQI), insomnia severity index (ISI); sinonasal outcome test 22 (SNOT-22); and study design. The exclusion criteria were: (1) studies not written in English; (2) case reports, reviews, conference abstracts, and letters; (3) studies with unclear and/or incomplete data; (4) pediatric studies (patients < 18 years of age), since CRSwNP in children exhibits distinct pathophysiological characteristics, lower prevalence, and higher rates of confounding systemic conditions such as cystic fibrosis and primary ciliary dyskinesia, which may independently affect sleep quality; additionally, sleep assessment instruments employed in the literature have not been uniformly validated for pediatric populations, and the pivotal trials establishing dupilumab’s efficacy in CRSwNP were conducted exclusively in adult populations; and (5) studies published before 2009 to discuss the solution of the last 15 years.

### 2.3. Data Extraction and Data Analysis

Two independent reviewers (A.M. and D.N.) performed the literature search and screened all records by title and abstract. The full texts of potentially relevant articles were then assessed for eligibility. Disagreements were resolved by discussion and consensus, with consultation of a third reviewer (E.D.C.) when necessary. Data from the included studies were extracted using a standardized form. The extracted variables included study characteristics (author, year, country, design), patient population, biologic agent, timing of biologic administration in relation to surgery, type and extent of surgery, comparator, outcomes assessed, duration of follow-up, and main findings. The risk of bias was assessed using tools appropriate for each study design. The methodological quality of the included studies was independently assessed by two reviewers. For non-randomized intervention studies, the ROBINS-I (Risk of Bias in Non-randomized Studies of Interventions) tool [9] was used to evaluate domains including bias due to confounding, participant selection, classification of interventions, and measurement of outcomes. For randomized trials, the Cochrane Risk of Bias (RoB 2) tool was applied [10].

### 2.4. Statistical Analysis and Summary of Findings

While Rodriguez-Iglesias et al. [7] recently performed a meta-analysis of global sinonasal outcomes (total SNOT-22 and NPS) in dupilumab-treated CRSwNP patients, a similar quantitative synthesis of sleep-specific outcomes was not possible in the present review. The reasons are threefold. First, the sleep domain of SNOT-22 is not uniformly extractable. Several included studies reported only total SNOT-22 scores without domain-level disaggregation. Second, the included studies employed heterogeneous sleep assessment instruments (ESS, PSQI, ISI, STOP-BANG, NOSE sleep domain) with different scoring metrics and minimal clinically important difference thresholds, precluding direct pooling. Third, reporting formats varied considerably (means with standard deviations, medians with interquartile ranges, or graphical representations without numerical precision). Consequently, a narrative synthesis was deemed the most appropriate and transparent approach, as prespecified in our methods. Therefore, the effects on individual outcomes and the overall quality assessments were described narratively. The authors of the included studies were not contacted for additional information.

### 2.5. Clarifying Clinical Relevance

To better contextualize the clinical relevance of the observed improvements in sleep quality, it is important to consider the minimal clinically important differences (MCID) for the measures used:

- SNOT-22 (sleep domain): The SNOT-22 is a 22-item questionnaire specifically designed to assess health-related quality of life in patients with chronic rhinosinusitis. Each item is scored from 0 (no problem) to 5 (as bad as it can be). The questionnaire can be divided into four validated domains [11], one of which is the sleep domain, comprising eight items: difficulty falling asleep, waking up at night, lack of a good night’s sleep, waking up tired, fatigue during the day, reduced productivity, reduced concentration, and frustrated/restless/irritated. The sleep domain score ranges from 0 to 40, with higher scores indicating greater sleep impairment. Although the MCID for the total SNOT-22 score is well established at 8.9 points [12], an improvement of 2–3 points per item in the sleep domain (on a 0–5 scale) can be considered clinically significant [6,7,13,14,15,16,17,18,19].
- PSQI: The PSQI assesses global sleep quality over a one-month interval. It includes 19 self-rated items grouped into seven component scores: subjective sleep quality, sleep latency, sleep duration, habitual sleep efficiency, sleep disturbances, use of sleep medication, and daytime dysfunction. Component scores are summed to yield a global PSQI score ranging from 0 to 21, with a score ≥ 5 indicating poor sleep quality. The MCID for the PSQI is 1.5 points [16,20].
- ESS: The ESS measures excessive daytime sleepiness across eight common situations (e.g., sitting and reading, watching television, driving). Each item is scored from 0 (no chance of dozing) to 3 (high chance of dozing), with total scores ranging from 0 to 24. A score > 10 indicates excessive daytime sleepiness suggestive of potential obstructive sleep apnea. The MCID for the ESS ranges from –2 to –3 points [16,21].
- ISI: The ISI evaluates the nature and severity of insomnia over the past two weeks. It consists of seven items assessing difficulty falling asleep, difficulty staying asleep, early-morning awakenings, sleep satisfaction, interference with daytime functioning, noticeability of impairment by others, and distress caused by sleep difficulties. Each item is scored from 0 to 4, with total scores ranging from 0 to 28. Scores < 7 suggest no relevant sleep disturbances. An MCD of 6 points in the ISI score is regarded as clinically significant [16,22].
- NOSE score: A change of 3.9–5.9 points on the overall NOSE score (0–100) is considered clinically significant, with scores < 25 suggesting mild obstruction [23]. However, no validated MCIDs were established for the NOSE score sleep domain (item 4).

In the studies examined, treatment with dupilumab generally produced improvements that exceeded these MCID thresholds, suggesting that the observed benefits are not only statistically significant but also clinically relevant for patients.

## 3. Results

**Figure 1:**
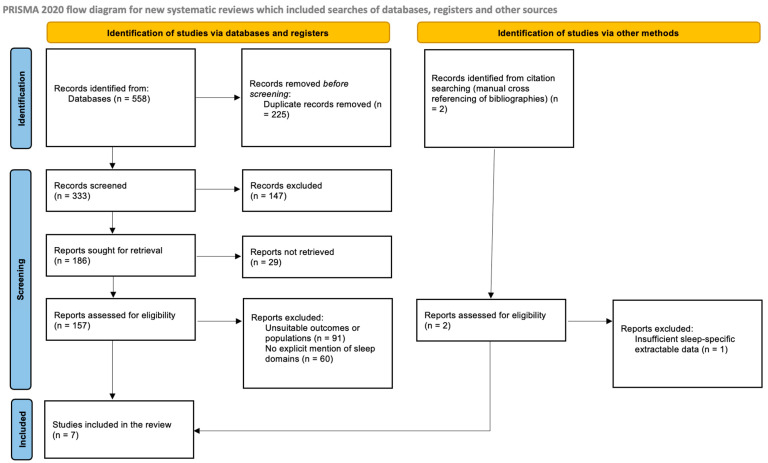
Prisma 2020 flow diagram [25] (Supplementary File S1). This work is licensed under CC BY 4.0. To view a copy of this license, visit https://creativecommons.org/icenses/by/4.0 (accessed on 23 July 2026).

The search criteria initially returned 558 articles. After removing 225 duplicate records, 333 articles were screened by title and abstract. Of these, 147 were excluded as irrelevant. The remaining 186 full-text reports were sought for retrieval, of which 29 could not be obtained. The 157 full-text reports assessed for eligibility underwent detailed evaluation. A total of 151 reports were excluded from the database search for the following reasons: unsuitable outcomes or populations (*n* = 91) and no explicit mention of sleep domains (*n* = 60). Two additional articles were identified through manual cross-referencing of bibliographies. Of these, one was included after full-text review, and the other was excluded due to insufficient sleep-specific extractable data. Ultimately, 7 articles met the inclusion criteria. The selection process is illustrated in Figure 1 (PRISMA 2020 flow diagram). The included studies involved 2164 patients, with a mean age of 51.67 years. The characteristics of these studies are detailed in Table 1, with further descriptions available in Table 2.

**Table 1:** Characteristics of the studies included.

Author (Year)	Country	Journal	Study Design	N. of Patients	Mean Age (Years)	Sex (M/F)	Mean Number of Surgeries	Patients with Asthma	Treatment Duration (Months)
Althomaly et al. (2024) [14]	Egypt	The Egyptian Journal of Otolaryngology	Cross-sectional study	15	39.9 ± 11.3	12/3	-	9	-
Busse et al. (2022) [15]	USA, UK, France	Journal of Asthma and Allergy	Post hoc analysis of two RCTs	1172	50	693/479	1.7	691	6 months (SINUS-24), 12 months (SINUS-52)
De Corso et al. (2023) [24]	Italy	Allergy	Multicenter retrospective study	648	54 (45–63)	400/248	2 (1–3)	366	12
Ferri et al. (2023) [16]	Italy	Annals of Allergy, Asthma and Immunology	Observational	29	54.0 ± 9.8	13/16	2.2 ± 1.7	24	3
Lang et al. (2026) [13]	Austria	Scientific Reports	Retrospective, longitudinal real-life cohort study	89	51.4 ± 14.1	51/38	2 (0–7)	57	6
Riva et al. (2024) [17]	Italy	American Journal of Otolaryngology–Head and Neck Surgery	Observational	45	57	29/16	-	26	12
Russo et al. (2025) [18]	Italy	American Journal of Rhinology and Allergy	Multicenter retrospective study	166	55.4 ± 13.4	104/62	-	-	12

**Table 2:** Summary of results.

Author	SNOT-22 Sleep Domain	ESS	ISI	PSQI	Sleep Disorders (VAS)	NOSE Sleep Domain	STOP-BANG
Althomaly et al. (2024) [14]	Baseline: 9 (6–13); post-treatment (6.7 months): 2 (0–2); *p* < 0.001	-	-	baseline: 6 (5–12); post-treatment (6.7 months): 5 (2–7); *p* < 0.04	-	baseline: 15 (5–15); Post-treatment (6.7 months): 0 (0–10); *p* < 0.001	
Busse et al. (2022) [15]	LS mean difference compared to placebo 6 months: −0.94 to −0.97 points (*p* < 0.001) 12 months: −1.01 to 1.05 points (*p* < 0.001)	-	.	-	-	-	
De Corso et al. (2023) [24]		-	-	-	Baseline: 6.0 (4.0–8.0); 1 month: 2.0 (0.0–4.0); 3 months: 1.0 (0.0–2.0); 6 months: 1.0 (.0.0–2.0); 9 months: 0.0 (0.0–2.0); 12 months: 0.0 (0.0–2.0) (*p* < 0.001)	-	
Ferri et al. (2023) [16]	Baseline: 12.1 ± 4.2; 1 month: 7.0 ± 4.9 (*p* < 0.001); 3 months: 6.8 ± 4.4 (*p* < 0.001)	Baseline: 7.9 ±4.5; 1 month: 6.17 ±4.8 (*p* < 0.001); 3 months: 4.3 ± 3.4 (*p* < 0.001)	Baseline: 13 ± 6.2; Month 1: 8.1 ± 5.7 (*p* < 0.001); Month 3: 7.0 ± 5.6 (*p* < 0.001)	Baseline: 9.2 ± 3.7; 1 month: 6.6 ± 3.6 (*p* < 0.001); 3 months: 6.4 ± 3.4 (*p* < 0.001)	-	-	High risk of sleep apnea at STOP-BANG in 31 (68.9%) and 1 (2.8%) patient at baseline and 1 month, respectively (*p* <0.001)
Lang et al. (2026) [13]	Baseline: 19.8 ± 10.4; 6 months: 9.5 ± 8.8 (*p* < 0.001)						
Riva et al. (2024) [17]	Baseline: 16 Month 3: 8 Month 6: 6 Month 12: 5 (*p* < 0.001)	Baseline: 4 Month 3: 2 Month 6: 1 Month 12: 1 (*p* < 0.001)	-	Baseline: 11 Month 3: 3 Month 6: 2 Month 12: 2 (*p* < 0.001)	-	-	
Russo et al. (2025) [18]	-	-	-	-	Baseline: 6 (4–8) 1 month: 4 (0.25–5) (*p* < 0.001) 3 months: 2 (0–3) (*p* < 0.001) 6 months: 0 (0–2) (*p* < 0.001) 12 months: 0 (0–2) (*p* = 0.004) 18 months: 0 (0–2) (*p* = 0.271) 24 months: 0 (0–2) (*p* = 0.654)		

Busse et al. [15] conducted a post hoc analysis of the SINUS-24 and SINUS-52 clinical trials to specifically evaluate the impact of dupilumab on sleep-related symptoms in CRSwNP patients, as detailed sleep quality outcomes were not a primary focus of the original trial publications. This analysis revealed that Dupilumab significantly improved sleep-related fatigue compared to placebo. At week 24, patients receiving dupilumab showed a mean reduction of 7.02 points in the SNOT-22 sleep domain, compared to a 1.53-point reduction in the placebo group (*p* < 0.001). Improvements were sustained at week 52, with a further reduction of 0.64 points in the dupilumab group compared to baseline (*p* = 0.02). Patients reported enhanced productivity and cognitive function, with a mean improvement of 1.46 points in concentration (*p* < 0.001) and 1.28 points in fatigue (*p* < 0.001) at week 24, which remained stable at week 52.

Ferri et al. [16] prospectively evaluated 29 patients with severe CRSwNP treated with dupilumab (300 mg every 2 weeks). Sleep-related outcomes were assessed at baseline, 1 month, and 3 months using validated questionnaires, including the ESS, ISI, PSQI, and sleep domain of the SNOT-22. The mean ESS decreased from 7.9 ± 4.5 at baseline to 6.17 ± 4.8 (*p* < 0.001) after one month, with further improvement to 4.3 ± 3.4 at 3 months (*p* < 0.001). Similarly, the ISI score improved from a baseline of 13.0 ± 6.2 to 8.1 ± 5.7 at 1 month (*p* < 0.001) and 7.0 ± 5.6 at 3 months (*p* < 0.001). Global PSQI scores, with a baseline mean of 9.2 ± 3.7 (consistent with poor sleep quality, as scores > 5 indicate poor sleep), decreased to 6.6 ± 3.6 at 1 month (*p* < 0.001) and 6.4 ± 3.4 at 3 months (*p* < 0.001). The proportion of patients reporting overall impaired sleep quality decreased from 93.1% at baseline to 44.8% at 3 months (*p* < 0.001).

De Corso et al. [24] evaluated 648 CRSwNP patients treated with dupilumab for up to 12 months. Sleep disturbances were assessed using a Visual Analogue Scale (VAS, 0–10). Median VAS sleep disorder scores improved rapidly and significantly from baseline (6.0, IQR: 4.0–8.0) to 1 month (2.0, IQR: 0.0–4.0), 3 months (1.0, IQR: 0.0–2.0), 6 months (1.0, IQR: 0.0–2.0), 9 months (0.0, IQR: 0.0–2.0), and 12 months (0.0, IQR: 0.0–2.0) (*p* < 0.001 for all timepoints). After 12 months, 96.9% of patients achieved a moderate-to-excellent clinical response per EPOS 2020 criteria [1], underscoring the sustained and clinically meaningful benefit of dupilumab on sleep quality.

Riva et al. [17] prospectively evaluated 45 CRSwNP patients treated with dupilumab over 12 months. Sleep outcomes were assessed using the PSQI, the sleep domain of the SNOT-22, and the ESS. Global PSQI scores improved from a baseline median of 11 (poor sleep quality) to 3 at 3 months, 2 at 6 months, and 2 at 12 months (*p* < 0.001). The SNOT-22 sleep domain decreased from a baseline median of 16 to 8 at 3 months, 6 at 6 months, and 5 at 12 months (*p* < 0.001). ESS scores improved from a baseline median of 4 to 2 at 3 months, 1 at 6 months, and 1 at 12 months (*p* < 0.001). Notably, the percentage of patients classified as having poor global sleep quality decreased dramatically from 88.9% at baseline to only 5.7% at 12 months.

Althomaly et al. [14] conducted a retrospective cross-sectional study including 15 CRSwNP patients treated with dupilumab. Sleep outcomes were assessed using the PSQI, the sleep domain of the SNOT-22, and the NOSE sleep domain, both at baseline and post-treatment (mean follow-up at 6.7 months). The median global PSQI score improved from 6 (IQR: 5–12) at baseline to 5 (IQR: 2–7) post-treatment (*p* = 0.04). The SNOT-22 sleep domain decreased from a median of 9 (IQR: 6–13) to 2 (IQR: 0–2) (*p* < 0.001). The NOSE sleep domain improved from 15 (IQR: 5–15) to 0 (IQR: 0–10) (*p* < 0.001). Before treatment, 53.3% of patients (8 of 15) reported poor sleep quality based on a global PSQI score > 5.

Lang et al. [13] conducted a retrospective real-life cohort study including 89 CRSwNP patients treated with dupilumab for six months. The median SNOT-22 sleep domain score improved significantly from 19.8 ± 10.4 at baseline to 9.5 ± 8.8 at six months (*p* < 0.001). All individual sleep-related items showed significant improvements: difficulty falling asleep decreased from 2 (IQR: 1–3) to 1 (IQR: 0–2) (*p* < 0.001); wake up at night decreased from 3 (IQR: 1–4) to 1 (IQR: 0–2) (*p* < 0.001); lack of a good night’s sleep decreased from 3 (IQR: 2–4) to 1 (IQR: 0–3) (*p* < 0.001); and wake up tired decreased from 3 (IQR: 2–4) to 1 (IQR: 0–2) (*p* < 0.001). The sleep domain demonstrated a large effect size (Cohen’s d = 1.1).

Russo et al. [18] conducted a multicenter retrospective study including 166 patients with severe uncontrolled CRSwNP treated with dupilumab for up to 24 months. Sleep disorders were assessed using a VAS (0–10). Median VAS sleep disorder scores improved significantly from baseline (6, IQR: 4–8) to 1 month (4, IQR: 0.25–5, *p* < 0.001), 3 months (2, IQR: 0–3, *p* < 0.001), and 6 months (0, IQR: 0–2, *p* < 0.001). Improvements were sustained at 12 months (0, IQR: 0–2, *p* = 0.004), with no further significant changes at 18 months (*p* = 0.271) and 24 months (*p* = 0.654). The most pronounced reduction in sleep disturbances occurred within the first six months of treatment.

### 3.1. SNOT-22 Sleep Domain Improvements Relative to MCID

The SNOT-22 sleep domain was the most consistently reported sleep-specific outcome across studies, with six of seven included studies reporting this parameter. When evaluated against the estimated MCID threshold of 2–3 points per item (approximately 4 points total for the 8-item sleep domain), a clear pattern emerges that distinguishes the post hoc RCT analysis from the observational studies.

The post hoc analysis of the SINUS-24 and SINUS-52 trials by Busse et al. reported the most modest improvements, with least squares mean differences compared to placebo of −0.94 to −0.97 points at 6 months and −1.01 to −1.05 points at 12 months, therefore lying below the estimated MCID threshold [15]. This analysis, while benefiting from the rigor of RCT design, did not report absolute baseline or post-treatment scores, limiting direct comparison with other studies. In contrast, all five observational studies reporting absolute SNOT-22 sleep domain scores demonstrated clinically meaningful improvements that met or exceeded the MCID threshold: Lang et al. [13] reported the most substantial improvement, with median scores decreasing from 12 (IQR: 1–3) at baseline to 1 (IQR: 0–3) at 6 months, representing an 11-point median reduction. The sleep domain demonstrated a large effect size (Cohen’s d = 1.1), with 76% of patients achieving the MCID of 4 points. Likewise, Riva et al. [17] documented progressive improvement from a baseline median of 16 to 8 at 3 months, 6 at 6 months, and 5 at 12 months, representing an 11-point reduction that was sustained over the 12-month follow-up period. Althomaly et al. [14] reported a median reduction of 7 points, from 9 (IQR: 6–13) at baseline to 2 (IQR: 0–2) post-treatment at a mean follow-up of 6.7 months. Ferri et al. [16] demonstrated improvement from a baseline mean of 12.0 ± 4.2 to 7.0 ± 4.9 at 1 month and 6.8 ± 4.4 at 3 months, representing reductions of 5.0 and 5.2 points above the estimated MCID threshold, respectively.

De Corso et al. [24] did not report the SNOT-22 sleep domain separately, instead using a VAS for sleep disorders, which showed rapid improvement from baseline median 6.0 to 1.0 at 3 months, sustained through 12 months.

### 3.2. Risk of Bias Assessment

**Figure 2:**
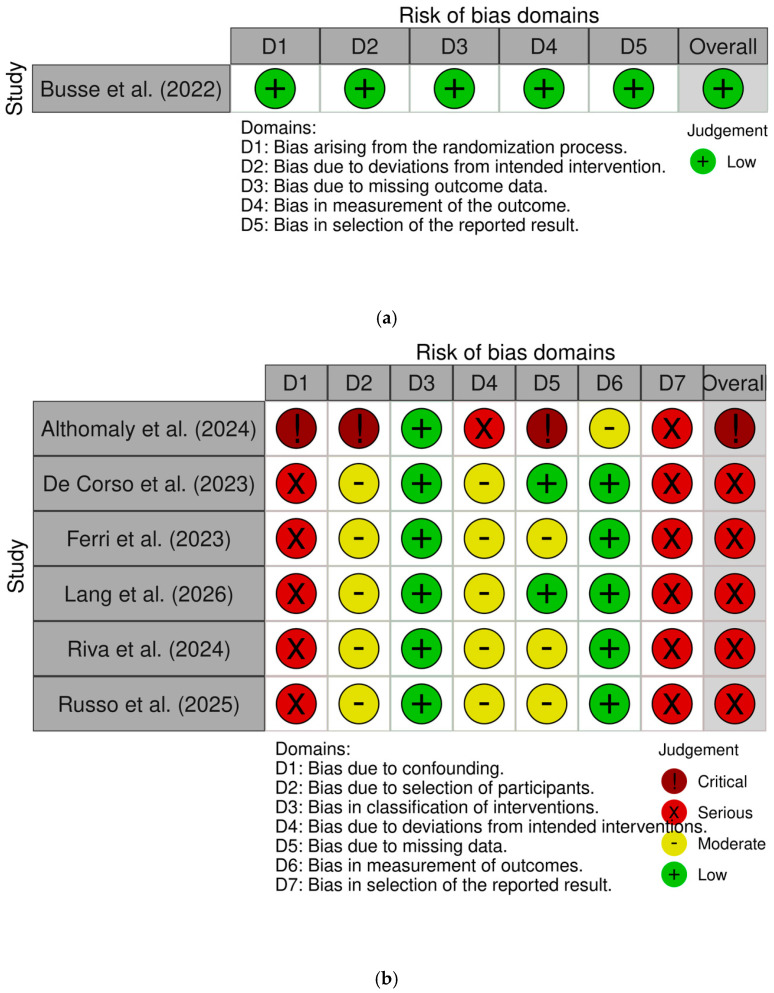
Risk of bias assessment. (**a**) Risk of bias assessment for randomized trials using the RoB 2 tool [15]; (**b**) Risk of bias assessment for non-randomized studies using the ROBINS-I tool [13,14,16,17,18,24].

The risk of bias for the included studies was assessed using the Cochrane RoB 2 tool for randomized trials and the ROBINS-I tool for non-randomized studies, and visually illustrated in Figure 2a,b with the use of the Risk-of-bias VISualization (robvis) tool [9,10,26].

Among the six observational studies, five were judged as having a serious overall risk of bias, primarily due to three interconnected issues [14,17,18,19,26]. First, unaddressed confounding represented a major concern: many patients with CRSwNP have comorbid conditions, such as asthma, OSA, and atopic dermatitis, that affect sleep quality alone. Without adequate adjustment for these confounders, observed improvements cannot be confidently attributed solely to dupilumab. Additionally, the potential for concomitant medication changes during the study periods further complicates causal attribution. Second, the lack of blinding in observational studies introduces the potential for placebo effects, which are particularly relevant for subjective outcome measures such as sleep questionnaires. Patient expectations regarding biologic therapy may have influenced responses on PROMs, potentially overestimating treatment effects. Third, selective reporting of sleep outcomes without preregistered protocols raises concerns about outcome reporting bias. Conversely, Althomaly et al. [14] was rated as having a critical risk of bias due to its cross-sectional design, small sample size (*n* = 15), and undefined treatment duration. The cross-sectional design provides the lowest level of evidence as it cannot establish a clear temporal relationship between treatment initiation and outcome assessment. On the other hand, Busse et al. [15] showed a low concern of bias as it was a post hoc analysis of two RCTs. However, the post hoc nature of the analysis introduces potential bias in outcome reporting, as sleep outcomes were not a prespecified primary or secondary endpoint in the original trial protocols. Additionally, the analysis did not report absolute baseline scores for the sleep domain, limiting direct comparison with other studies and precluding assessment of the clinical significance of the observed changes relative to MCID.

### 3.3. Comparison with Other Systematic Reviews and Meta-Analyses

It is worth comparing our findings to the recent meta-analysis by Rodriguez-Iglesias et al. [7], which evaluated sinonasal outcomes (SNOT-22 total and NPS) from dupilumab real-world studies. Their reported mean SNOT-22 total score reduction of 37.2 points (exceeding the MCID of 8.9 points by a factor of 4.18) provides important context for interpreting our sleep domain findings. In our review, the SNOT-22 sleep domain showed mean reductions of up to −7.02 points [15] and −9 points [14], suggesting that the sleep-related benefits constitute a meaningful component of the overall quality-of-life improvement. Importantly, while their meta-analysis could not isolate sleep-specific effects due to heterogeneous outcome reporting, our systematic review addresses this precise gap by focusing on studies that explicitly reported validated sleep measures.

Our findings can be further contextualized by comparison with the meta-analysis by Fried et al. [6], who investigated the effect of conventional CRS treatments (endoscopic sinus surgery, intranasal, and systemic corticosteroids) on sleep quality. They observed clinically meaningful improvements in patient-reported sleep outcomes, including the ESS (mean difference −2.8), Pittsburgh Sleep Quality Index (mean difference −2.4), and the SNOT-22 sleep domain (mean difference −5.4). However, no corresponding changes in objective polysomnographic parameters (AHI, oxygen nadir) were observed, suggesting that the subjective sleep benefits observed in CRS patients may be driven primarily by improvements in nasal patency and reduction of bothersome symptoms (e.g., congestion, rhinorrhea, post-nasal drip) rather than by objective resolution of sleep-disordered breathing events.

## 4. Discussion

This systematic review evaluated the impact of dupilumab on sleep quality in patients with severe CRSwNP. Across seven included studies comprising 2164 patients, dupilumab was consistently associated with improvements in patient-reported sleep outcomes. The most robust evidence was observed for the SNOT-22 sleep domain, with five of six studies reporting clinically meaningful improvements exceeding the estimated MCID threshold. Improvements were also observed across other sleep-specific measures, including the PSQI, ESS, ISI, and VAS for sleep disorders. The most substantial improvements occurred within the first 3–6 months of treatment and were sustained through 12–24 months of follow-up, where assessed. Patients with more severe baseline sleep impairment appeared to derive the greatest benefit.

### 4.1. What Do We Know About the Relationship Between CRSwNP and Sleep?

Cytokines, such as IL-1, IL-4, IL-6, IL-10, and IL-13, are released locally in CRSwNP and have been linked to sleep regulation through their central effects.

Interestingly, IL-4 has been shown to play a critical role in higher brain functions, including sleep, memory, and learning [27]. High levels of interleukin (IL)-1β, IL-4, and IL-10 have been shown to increase latency to Rapid Eye Movement (REM) sleep, shorten the duration of REM sleep, and decrease latency to sleep onset [28].

Likewise, IL-13 has inhibited spontaneous sleep, especially non-REM sleep, in experimental settings [29], suggesting that this cytokine might play an additional role in sleep disturbance in patients with CRSwNP. The upregulation of IL-4 and TGF-β may contribute to inflammatory brain-mediated effects on sleep quality, whereas IL-13 may be a pleiotropic signaling molecule influencing sleep, QOL, and CRS disease severity [30]. Endogenous substances moderating the effects of IL-1 and TNF include anti-inflammatory cytokines such as IL-4, IL-10, and IL-13. Clinical conditions altering IL-1 or TNF activity are associated with changes in sleep, for example, infectious disease and sleep apnea [31].

### 4.2. How Can We Explain the Improvement in Sleep Quality in CRSwNP Patients Receiving Dupilumab?

The improvement in sleep quality observed in CRwNP patients is probably attributable to the interaction of dupilumab with type 2 inflammatory pathways. First of all, dupilumab blunts nasal inflammation by reducing eosinophilic infiltration and mucosal edema, resulting in decreased nasal congestion and improved airflow dynamics [16,17]. This reduction in upper airway obstruction correlates with improved nocturnal breathing and subsequently reduced sleep fragmentation, as nasal congestion has proved to be a prime mover of sleep disturbances in CRSwNP in OSA [24,32,33]. Moreover, the systemic anti-inflammatory properties of dupilumab may contribute to ameliorating sleep quality. By reducing levels of systemic inflammation, dupilumab positively impacts fatigue and daytime sleepiness [16,17,34]. Interestingly, a subsequent study by Alt et al. [30] postulated the existence of a specific somnogenic inflammatory airway phenotype in patients with CRSwNP and sleep dysfunction, characterized by elevated levels of IL-4, IL-5, and IL-13, along with hypereosinophilia. The study highlighted that the inflammatory milieu in CRSwNP may not only contribute to local effects, such as nasal obstruction, but may even disrupt sleep architecture through systemic effects on the central nervous system, further exacerbating sleep-related symptoms. As highlighted by Kubota et al. [29], IL-13 suppresses the production of IL-1β and TNF-α in vitro. Since IL-1β and TNF-α are known sleep regulators, blocking IL-13 could alter these pathways.

### 4.3. Does Dupilumab Improve Sleep Quality Alone, or Are There Any Objective Improvements in OSA?

While dupilumab significantly improves sleep quality, its direct impact on polysomnographic parameters remains unknown. Indeed, the nasal cavity is a primary site of airflow resistance, and its obstruction may lead to increased negative respiratory pressure, fostering pharyngeal collapse during sleep [35,36]. Therefore, improving nasal patency through surgical and nonsurgical interventions contributes to upper airway stabilization, thereby decreasing the frequency of apneas. However, nasal treatment alone is rarely curative for OSA as it primarily targets nasal resistance and may not address multilevel airway collapse, underscoring the importance of a comprehensive, patient-specific treatment approach [37]. Although there is a potential link underlying the aetiology of CRSwNP and OSA, since both are characterized by dysregulated airway inflammation and structural remodeling, the evidence supporting dupilumab’s ability to reduce apnea events is still limited. Up to 64.7% of patients diagnosed with CRSwNP appeared to have comorbid OSA, far exceeding the prevalence rate of OSA in the general population (8–38%) [32]. Interestingly, these findings delineate a different phenotypic asset of OSA patients, who appear less obese than non-comorbid patients [32]. Indeed, the shared presence of pro-inflammatory cytokines in both conditions suggests that the anti-inflammatory properties of dupilumab may directly improve OSA severity through its local and systemic effects on airflow [8,38]. Notably, the relationship between OSA severity and the extent of CRSwNP appears to persist also in non-type 2 inflammatory phenotypes, such as in the non-eosinophilic CRSwNP [39]. However, the magnitude of sleep disturbances is greater in type 2-driven CRSwNP, further emphasizing the importance of targeting type 2 inflammation to improve sleep quality [39]. This aligns with the findings of De Corso et al. [24], who reported that dupilumab significantly improved QoL and sleep-related outcomes in patients with severe CRSwNP, particularly in those with a type 2 inflammatory phenotype.

### 4.4. What Are the Other Conditions Associated with Type 2 Inflammation That Can Impair Sleep Quality?

On top of CRSwNP, other type 2 inflammatory-driven conditions, such as asthma, atopic dermatitis, and eosinophilic esophagitis, may contribute to sleep fragmentation [39,40]. For instance, asthma is tightly linked to type 2 inflammation and may cause nocturnal symptoms such as wheezing, coughing, and shortness of breath, resulting in frequent awakenings and fragmented sleep [33]. Therefore, the improved asthma control provided by dupilumab may indirectly enhance sleep quality [15]. Likewise, atopic dermatitis is characterized by intense nocturnal pruritus, prompting the patients to awaken at night, ultimately disrupting sleep structure frequently. Since a type 2 inflammatory phenotype also subtends atopic dermatitis, it is not surprising that treatment with dupilumab has been shown to alleviate itching and improve sleep in these patients [33,41,42]. Lastly, eosinophilic esophagitis is another condition belonging to the spectrum of type 2 inflammation, which can cause dysphagia, chest pain, or even food impaction, which may cause nocturnal discomfort or even impact sleep due to heightened levels of choking-driven anxiety [43,44]. Indeed, Dupilumab is emerging as a promising option for treating eosinophilic esophagitis, potentially improving sleep quality in affected patients [33].

### 4.5. The MCID Threshold: Heterogeneity in Treatment Response

Our analysis reveals a striking pattern: the post hoc RCT analysis by Busse et al. [15] demonstrated improvements below the estimated MCID (−0.94 to −1.05 points), while the observational studies consistently demonstrated clinically meaningful improvements that substantially exceeded the MCID. This divergence is particularly noteworthy given that the SNOT-22 sleep domain was the most consistently reported outcome measure across studies, with six of seven included studies reporting it. The most robust evidence for clinically meaningful improvement comes from the observational studies with longer follow-up and more severe baseline disease. For instance, Lang et al. [13] reported an 11-point median reduction with a large effect size, while Riva et al. [17] demonstrated progressive improvement over 12 months, with the 11-point reduction maintained throughout the follow-up period.

Several factors may explain the more modest improvements observed in the Busse et al. analysis. First, the post hoc analysis was derived from a population selected for clinical trials, which may have excluded patients with the most severe sleep disturbances due to exclusion criteria related to comorbidities, medication use, or disease severity. Second, the trial population may have had lower baseline sleep domain scores, limiting the potential for large absolute improvements. Third, the least squares mean difference from placebo, while statistically robust, may not capture the full magnitude of improvement experienced by individual patients. Fourth, the 6- to 12-month follow-up in the RCTs may have been insufficient to capture the full trajectory of sleep improvement, as the observational studies continued to show improvement through 12 months. The observational studies demonstrating clinically meaningful improvements also have limitations. The lack of blinding, potential for confounding, and absence of objective sleep measures may have contributed to the overestimation of treatment effects. However, the consistency of the findings across multiple observational studies from different centers and countries provides some reassurance that the observed benefits are genuine. Furthermore, the dose–response relationship, with greater improvements observed in patients with more severe baseline sleep impairment, adds biological plausibility to the findings.

### 4.6. Limitations and Future Perspectives

Despite these encouraging results, several important limitations must be acknowledged. First, the absence of polysomnographic evaluations in all included studies precludes objective assessment of changes in sleep architecture, apnea–hypopnea index, or nocturnal oxygen desaturation. While Patient-Reported Outcome Measures (PROMs) provide valuable information on patients’ subjective perceptions of sleep, Polysomnography remains the gold standard for objectively assessing sleep quality, sleep architecture, and sleep-related breathing disorders. Without these objective data, we cannot determine whether dupilumab improves specific parameters such as sleep efficiency, sleep latency, sleep stage distribution, or the AHI. Second, the majority of observational studies were rated as having a serious risk of bias (ROBINS-I), primarily due to unaddressed confounding and selective reporting. Third, the post hoc nature of the only RCT-derived analysis introduces potential bias in outcome reporting. Fourth, a quantitative synthesis was not feasible due to heterogeneity in outcome measures, follow-up durations, and data reporting formats. Fifth, no comparative head-to-head studies (dupilumab vs. other biologics or vs. conventional therapies). This methodological limitation, which we acknowledge as a gap in the field, highlights the need for standardized reporting of sleep outcomes in future dupilumab studies. Lastly, potential overlap among Italian cohorts cannot be entirely excluded, as several studies were conducted in the same country during partially overlapping time periods, and individual patient identifiers were not available to verify uniqueness. This represents a potential source of bias in the aggregate evidence.

For future studies on CRSwNP and sleep quality, we recommend adopting a standardized set of outcome measures, including: (1) the PSQI as a primary measure of sleep quality; (2) the ESS for daytime sleepiness; (3) the sleep domain of SNOT-22; and (4) objective sleep measures through polysomnography or actigraphy. The adoption of these standardized metrics would facilitate more meaningful comparisons between studies and enable more robust meta-analyses in the future.

## 5. Conclusions

Based on the findings of this systematic review, dupilumab was associated with significant improvements in patient-reported sleep quality across seven included studies, including reductions in PSQI, ESS, ISI, and SNOT-22 sleep domain scores. However, the evidence base has substantial limitations. All observational studies were rated as having serious or critical risk of bias; no study included objective sleep measures such as polysomnography, and a meta-analysis was not feasible due to heterogeneity in outcome measures and reporting styles. No head-to-head studies comparing dupilumab with other biologics or conventional therapies for sleep-specific outcomes are available. Therefore, while dupilumab may be a reasonable therapeutic option for CRSwNP patients with refractory sleep disturbances, this recommendation is based on low-certainty evidence. High-quality RCTs with prespecified sleep endpoints and polysomnographic assessments are needed before definitive conclusions can be drawn.

## Data Availability

No new data were created in the making of this article.

## Supplementary Materials

The following supporting information can be downloaded at: https://www.mdpi.com/article/10.3390/jcm15156010/s1, Supplementary File S1: PRISMA 2020 Checklist [25].

## Abbreviations

The following abbreviations are used in this manuscript:

AHI	Apnea–Hypopnea Index
CRSwNP	Chronic Rhinosinusitis with Nasal Polyps
CRSsNP	Chronic Rhinosinusitis without Nasal Polyps
EPOS	European Position Paper on Rhinosinusitis
ESS	Epworth Sleepiness Scale
EUFOREA	European Forum for Research and Education in Allergy and Airways diseases
FDA	US Food and Drug Administration
FeNO	Fractional Exhaled Nitric Oxide
HRQoL	Health-Related Quality of Life
IgE	Immunoglobulin E
IL-4	Interleukin-4
IL-4Rα	Interleukin-4 receptor alpha
IL-5	Interleukin-5
IL-10	Interleukin-10
IL-13	Interleukin-13
INCS	Intranasal Corticosteroids
ISI	Insomnia Severity Index
MCID	Minimal Clinically Important Difference
MeSH	Medical Subject Headings
NOSE	Nasal Obstruction Symptom Evaluation
NPS	Nasal Polyp Score
NREM	Non-Rapid Eye Movement
OSA	Obstructive Sleep Apnea
PRISMA	Preferred Reporting Items for Systematic Reviews and Meta-Analyses
PROMs	Patient-Reported Outcome Measures
PSQI	Pittsburgh Sleep Quality Index
QoL	Quality of Life
REM	Rapid Eye Movement
ROBINS-I	Risk of Bias in Non-randomized Studies of Interventions
RoB 2	Cochrane Risk of Bias tool for randomized trials
SDB	Sleep-Disordered Breathing
SNOT-22	Sinonasal Outcome Test-22
TNF	Tumor Necrosis Factor
TGF-β	Transforming Growth Factor beta
VAS	Visual Analogue Scale
